# Shared Genetic Contribution of Type 2 Diabetes and Cardiovascular Disease: Implications for Prognosis and Treatment

**DOI:** 10.1007/s11892-018-1021-5

**Published:** 2018-06-25

**Authors:** Rona J. Strawbridge, Natalie R. van Zuydam

**Affiliations:** 1 College of Medical, Veterinary and Life Sciences, University of Glasgow, Room 113, 1 Lilybank Gardens, Glasgow, G12 8RZ UK; 2 Cardiovascular Medicine, Department of Medicine Solna, Karolinska Institutet, Stockholm, Sweden; 3 Wellcome Centre Human Genetics, University of Oxford, Roosevelt Drive, Headington, Oxford, Oxfordshire, OX3 7BN UK; 4 Oxford Centre for Diabetes Endocrinology and Metabolism, Churchill Hospital, Headington, Oxford, Oxfordshire, OX3 7LE UK

**Keywords:** Type 2 diabetes, Coronary artery disease, Ischemic stroke, Peripheral artery disease, Risk factors, Genetics; Mendelian randomisation

## Abstract

**Purpose of Review:**

The increased cardiovascular disease (CVD) risk in subjects with type 2 diabetes (T2D) is well established. This review collates the available evidence and assesses the shared genetic background between T2D and CVD: the causal contribution of common risk factors to T2D and CVD and how genetics can be used to improve drug development and clinical outcomes.

**Recent Findings:**

Large-scale genome-wide association studies (GWAS) of T2D and CVD support a shared genetic background but minimal individual locus overlap.

**Summary:**

Mendelian randomisation (MR) analyses show that T2D is causal for CVD, but GWAS of CVD, T2D and their common risk factors provided limited evidence for individual locus overlap. Distinct but functionally related pathways were enriched for CVD and T2D genetic associations reflecting the lack of locus overlap and providing some explanation for the variable associations of common risk factors with CVD and T2D from MR analyses.

## Introduction

It is well established that cardiovascular disease (CVD) and type 2 diabetes (T2D) are concomitant. Subjects with T2D are at 3–4 × higher risk of developing CVD and many of the risk factors are shared between the two diseases, particularly hyperglycaemia, obesity, haemodynamic disturbances and dyslipidaemia [1]. Because the pathologies of both complex diseases begin long before the clinical presentation, epidemiology has been unable to answer the question of whether there are shared pathological mechanisms between CVD and T2D. There is also epidemiological evidence that CVD presents differently in subjects with T2D compared to those without diabetes. Generally, there is a higher atherosclerotic burden [2]; peripheral arterial disease (PAD) presents distally [3]; and T2D increases the risk of specific ischaemic stroke subtypes [4]. Whether these differences in CVD manifestation represent common mechanisms between CVD and T2D, or whether T2D has a disruptive influence on pathways related to the vasculature and haemodynamic factors remains largely unknown.

Experimental animal models for T2D and CVD have been developed, although these have limited utility as they typically involve a single gene alteration (in contrast to the evidence of polygenicity in humans), and the disease pathology and biochemical profiles do not resemble those observed in humans [5]. Large-scale genome-wide associations studies (GWAS) have provided robust evidence for many genetic variants influencing various aspects of metabolic and cardiovascular disease [6•, 7–10]. Cardiovascular disease, T2D and their shared risk factors are heritable and genetic information have been used in different ways to identify tissues, genes and pathways relevant to CVD in the context of T2D. Genetic data has also been used to separate factors on the causal pathway to CVD in subjects with T2D from those that are correlated with the disease. This review focuses on the current understanding of the shared genetic contributions to CVD and T2D, mechanistic insights from Mendelian randomisation (MR) and pathway analyses, and how these may affect drug development and clinical practice in the future.

### Shared Genetic Background Amongst Cardiometabolic Traits—Looking Genome-Wide

**Fig. 1:**
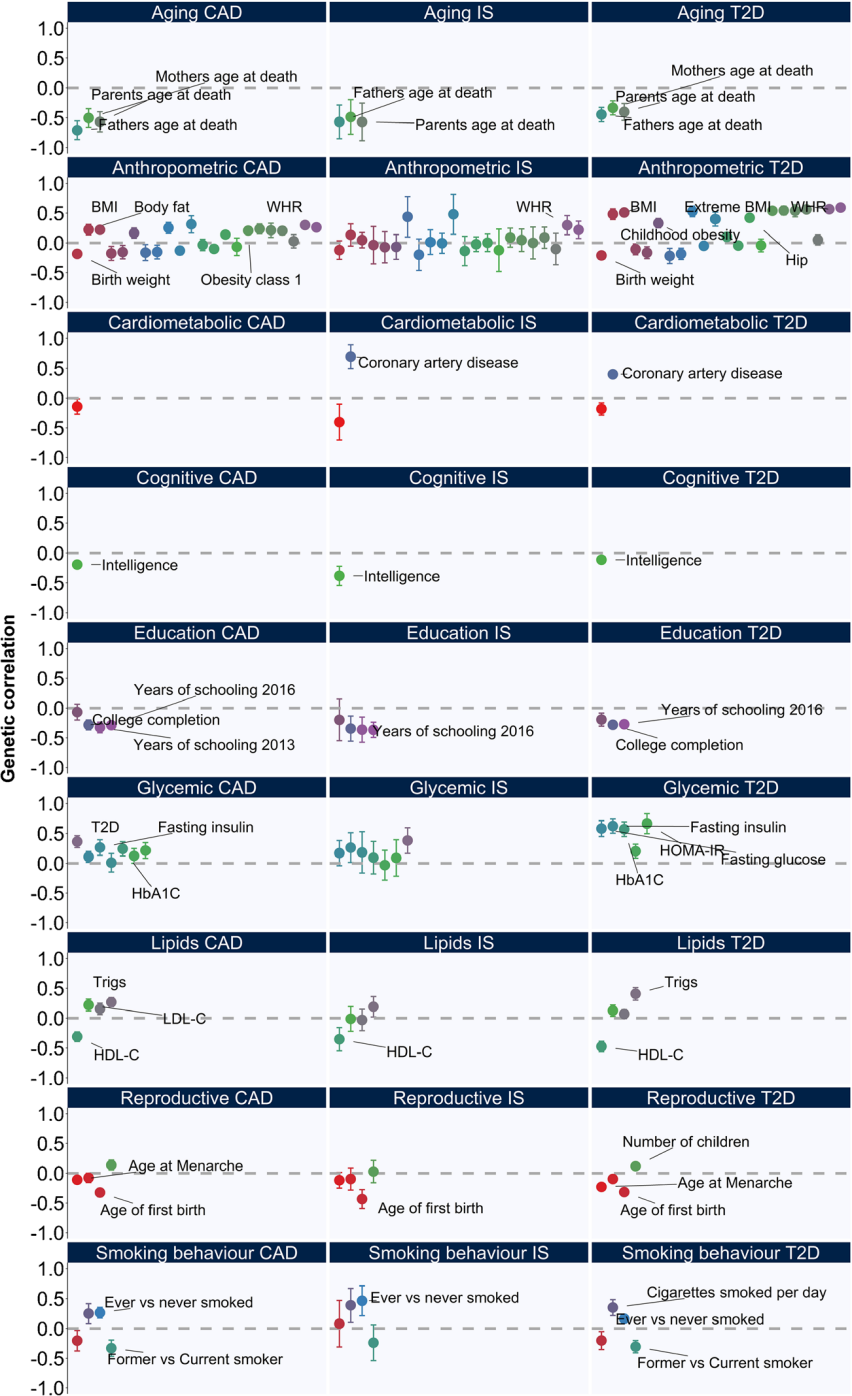
Genetic correlation analyses with 120 traits available from LD hub showed that type 2 diabetes, coronary artery disease and ischaemic stroke share a genetic background with each other but also with other traits. Some of the traits that passed *p* ≤ 4.2 × 10^−4^ (0.05/120) are labelled. Coronary artery disease (CAD); ischaemic stroke (IS); type 2 diabetes (T2D); body mass index (BMI); waist-hip ratio (WHR); triglycerides (trigs); low-density lipoprotein cholesterol (LDL-C); high-density lipoprotein cholesterol (HDL-C)

Shared genetic correlation can be determined by comparing allelic effects, genome-wide, between two traits or diseases. If there is a correlation between allelic effects, then a measure of shared genetic background can be derived [11]. Large GWAS of T2D [6•], coronary artery disease (CAD) [7] and ischaemic stroke [10] have been used to assess the shared genetic background between CVD and T2D using all variants included in the GWAS [12•]. Genome-wide, there is strong evidence that CAD (rg[se] = 0.40[0.03], *p* = 2.5 × 10^−46^) [6•] and ischemic stroke (rg[se] = 0.38[0.11], *p* = 5.0 × 10^−4^) share a genetic background with T2D, where variants associated with increased risk of CVD are also associated with increased risk of T2D (Fig. 1). These analyses indicate a shared genetic background for CVD and T2D but not which mechanisms are represented by the genetic variants contributing to the overlap or the direction of effect.

There is also evidence that CVD and T2D share a genetic background with common risk factors based on analyses of 120 traits available from LD hub (Fig. 1) [12•]. CVD and T2D were positively genetically correlated with each other, showing a shared genetic background. Traits related to parents’ age at death, years’ of schooling and age of first birth, were negatively correlated with T2D and CVD. Genetic variability associated with increased obesity, fasting insulin, glycated haemoglobin, triglycerides and propensity to become a smoker were also positively genetically correlated with T2D, CAD and ischaemic stroke (Fig. 1). These analyses show that CVD and T2D have a shared genetic background with each other and also with their common risk factors. Efforts to disentangle the complex relationship between T2D and CVD and their shared risk factors have included pathway analyses, GWAS and MR analyses.

## Cardiovascular Disease and Type 2 Diabetes Have Pathways in Common but Not Genes

Evaluating the tissue-specific effects of T2D and CVD associated loci is key to understanding how overlapping and functionally related pathways contribute to the development of CVD and T2D and how the effect of individual loci may differ by disease context. This requires integration of GWAS data with tissue-specific expression quantitative trait loci (eQTL) data and chromatin states [6•, 13–15]. There have been few studies of overlapping pathways between CVD and T2D, and these have been relatively small [16].The largest of these studies [17] integrated multi-ethnic GWAS of T2D and CVD with tissue-specific eQTL data and co-expression networks to identify genes and pathways enriched for T2D and CVD genetic associations. Co-expression networks are constructed from genes that have similar expression patterns as co-expressed genes are more likely to be functionally related. Co-expression networks can be annotated with pathway data, and GWAS data overlaid, to identify which co-expression networks and pathways are enriched for genetic associations.

Co-expression networks related to carbohydrate metabolism and glycan degradation were enriched in T2D and CVD but by non-overlapping genetic association signals. Other co-expression networks enriched for T2D genetic association signals were distinct from those enriched for CVD signals but were functionally related to specific pathways: lipid and fatty acid metabolism; glucose metabolism; oxidation; and cytokine signalling [17]. These analyses indicate sparse direct overlap of genes and pathways contributing to T2D and CVD but show that different genes are likely to be important in a set of common pathways relevant to the development of T2D and CVD. While pathway analysis shows that there is limited overlap in processes contributing to both diseases, it does not indicate whether these processes affect the development of T2D and CAD in the same way.

## Few Loci Jointly Contribute to Type 2 Diabetes and Cardiovascular Disease

Large GWAS of CVD in the context of T2D have investigated the presence of overlapping loci by (1) conducting GWAS of individual traits and looking for overlap, analysing CVD and T2D in a combined GWAS, and analysing CVD stratified by T2D status. Based on individual GWAS studies of T2D [6•], CAD [7], large artery stroke [10] and PAD [9], there are overlapping signals of association in the *CDKN2A/B* locus (Table 1). Variants in this locus have been associated with CAD severity in subjects with T2D, but this study was small [18], and larger studies of CAD stratified by T2D status have shown no difference in allelic effects by T2D status in this region, indicating that this locus is associated with CAD irrespective of T2D status [19]. A combined GWAS of T2D and CAD identified a single variant associated with both T2D and CAD at genome-wide significance (*p* ≤ 5 × 10^−8^, a threshold used in GWAS to identify robust associations) near *IRS1* (Table 1). The study describes eight additional lead variants from eight loci that are associated with T2D and CAD, but these are not genome-wide significant for both diseases. They share the same risk allele for seven lead variants and opposite risk alleles for *APOE* [20•]. This highlights that even in large studies there are few overlapping CVD and T2D loci, that few variants contribute jointly to CVD and T2D, and that these variants do not always share the same risk allele for T2D and CVD.

**Table 1:** There are two loci with evidence of association signal overlap amongst cardiovascular disease and type 2 diabetes based on large genome-wide association studies

Chr:Pos (b37)	SNP (gene)	Phenotype	EA (EAF)	OR (95%CI)	*p*
9:22043612	rs1412830 (*CDKN2A/B*)	Type 2 diabetes	C (0.63)	1.04 (1.02–1.05)	9.1 × 10^−8^
		Coronary artery disease	C (0.68)	1.12 (1.10–1.15)	2.6 × 10^−30^
		Peripheral artery disease	C (0.63)	1.06 (1.03–1.09)	7.6 × 10^−4^
		Large vessel stroke	C (0.63)	1.16 (1.08–1.26)	1.5 × 10^−4^
2:227020653	rs7578326 (*IRS1*)	Type 2 diabetes	A (0.65)	1.07 (1.05–1.09)	2.3 × 10^−13^
		Coronary artery disease	A (0.65)	1.05 (1.04–1.07)	4.7 × 10^−10^

Smaller studies have reported loci associated with CVD in the context of T2D. A study of CAD in subjects with T2D reported an association near *GLUL* with CAD that showed some evidence for interaction with T2D status [21]. This finding was not supported by a larger study of CAD in subjects with T2D that found no variants specifically associated with CAD in the context of T2D [19]. The Action to Control Cardiovascular Risk in Diabetes (ACCORD) trial reported two variants associated with fatal cardiovascular events in subjects under intense glycaemic control. Despite large-scale efforts to identify overlapping genomic regions, there are few robustly identified loci. However, most T2D risk-raising alleles are also risk-raising alleles for CAD [20•] and MR studies have shown a causal link between T2D and CAD [20•].

### A Causal Relationship Between Type 2 Diabetes and Cardiovascular Disease

MR analyses deconvolute the causal relationship between two traits where the relationship may be confounded by environmental effects. In contrast to genetic correlation, which assesses the shared genetic background of two traits across all variants in a GWAS, MR uses genetic risk scores (GRS) of SNPs as a genetic instrument to test the causal effects of one trait on another. Since unlinked genotypes are randomly allocated at birth, the association between genetic variation determining one trait and the genetic variation determining another is free from environmental confounding. Bidirectional MR analyses can be used to distinguish between biomarkers that are on the causal pathway to a disease from those that are a consequence of a disease—otherwise known as reverse causation, such as the relationship between C-reactive protein (CRP) and CAD [22]. There are several assumptions that must be met for MR analyses to be valid. One is that the genetic instrument should only represent the effect of the trait being tested. Variants can have pleiotropic effects, where they influence more than one trait, for example CRP and low-density lipoprotein cholesterol (LDL-C) levels [23]. If this pleiotropy is not taken into account, this can lead to spurious associations between a trait GRS and an outcome [24].

Variants associated with T2D have been shown to contribute to the development of the disease through different mechanisms, such as beta cell function, obesity, insulin secretion, obesity and hyperlipidaemia [6•, 15]. MR analyses of T2D and CVD have been conducted during different epochs of T2D locus discovery. Early studies, that did not take variant pleiotropy (associations with other cardiometabolic traits) into account, found associations between a T2D-GRS and CAD [25, 26] and separately with ischaemic stroke (large artery and small vessel stroke only) [27]. However, the average CAD risk per T2D allele was lower than expected [25] indicating that the GRS did not account for the all the risk of CAD observed in subjects with T2D. More recent MR studies have generated GRS based on more T2D-associated variants and have leveraged pleiotropic variant associations to construct T2D-GRS that contain variants associated with other similar traits. T2D-GRS constructed of non-pleiotropic T2D-associated variants was associated with CAD [28], but those constructed from variants pleiotropic for established CAD risk factors and for glycaemic traits have shown variable associations with CAD.

MR studies of shared risk factors for T2D and CVD have shown variable association with either T2D and CVD. There is variable evidence to support a role for high-density lipoprotein cholesterol (HDL-C), LDL-C, triglycerides, obesity, adiponectin and hyperglycaemia in the development of T2D and CVD [29–33]. Genetic variability increasing levels of LDL-C and triglycerides have been associated with increased risk of CAD but decreased the risk of T2D [34]. This is juxtaposed to the literature that correlates increased serum levels of LDL-C and triglycerides with increased T2D risk [35] Genetic variability increasing HDL-C levels was shown to be protective of CAD and T2D [34, 36], but other studies that excluded pleiotropic variants found no effect of HDL-C on CAD or T2D risk [37, 38].

Genetic determinants of glycaemic traits have been studied in healthy populations, which do not necessarily represent the genetic variability that determines glycaemic traits in subjects with T2D [8]. Some MR studies have tried to address this through careful selection of variants used to build genetic instruments for glycaemic traits. Genetic instruments consisting of T2D-associated variants that were also associated with any other glycaemic trait were not associated with CAD [20•]. However, GRS constructed from T2D associated variants also associated with HbA1c, beta cell function and insulin resistance were respectively associated with increased CAD risk but not those associated with fasting glucose and T2D [26]. On the other hand, instruments for fasting glucose that exclude T2D-associated variants were associated with increased CAD risk [28]. This suggests, perhaps unsurprisingly, that genetic mechanisms and underlying pathways that increase the risk of T2D do not uniformly influence CAD risk.

Results from MR studies can vary despite testing the relationship between the same traits and outcomes. This could reflect between-study differences, such as GRS strength, choice of variants included in the GRS, the sample size of the study, how subjects were recruited and the study design. Study design and participation can introduce collider bias creating false causal relationships between two traits [39, 40]. In cross-sectional studies, this can be introduced by selection bias. If subjects with the highest genetic risk of a disease are less likely to participate in a study then this may cause an inverse association between GRS for known risk factors and the disease outcome [39]. In MR studies of disease progression in cases only, factors that are causal for disease onset may also be associated with disease progression through association with confounders of disease incidence and progression. Overall, MR studies have highlighted the complex relationships amongst T2D, CVD and their shared risk factors and how these are challenging to deconvolute.

### Epigenetic Changes and Hyperglycaemia

Epigenetic changes to gene expression usually involve histone modifications and DNA methylation in response to a stimulus, such as disease state or environment. There is some experimental evidence of epigenetic changes that modify the risk of CVD, induced by shared risk factors for CVD and T2D, including hyperglycaemia [41]. In the Diabetes Control and Complications Trial (DCCT) and follow-up Epidemiology of Diabetic Complications and Interventions Trial (EDIC), intensive glycaemic control was shown to reduce the progression of complications (including cardiovascular) in subjects with T1D, but not completely abrogate them [42]. The concept of ‘metabolic memory’ was coined to account for the observation, which has been shown to occur because of epigenetic changes induced by hyperglycaemia [43]. Hyperglycaemia has been shown to play a causal role in the development of T2D and CVD [30], although trials of intensive glycaemic control in subjects with T2D have demonstrated variable effects on rates of CVD and all-cause mortality.

A large meta-analysis of glycaemic intervention trials (34,533 subjects) showed a small reduction in non-fatal myocardial infarction in the glucose-lowering group, but no overall effect on all-cause mortality or CVD death [44]. Two of the largest glycaemic intervention trials were conducted for 5 years or less [45, 46], and may have been too short to observe effects on cardiovascular outcomes and all-cause mortality [47]. After 5 years of follow-up in the Action in Diabetes and Vascular Disease (ADVANCE) trial, there was a non-significant trend of reduced CVD and all-cause mortality in the treatment arm, and in a 10-year follow-up of the United Kingdom Prospective Diabetes Study (UKPDS), there were significantly reduced rates of CVD and all-cause mortality in the treatment intervention groups [47]. A reduction in myocardial infarction and all-cause death was observed in the 10-year follow-up, despite the loss of glycaemic differences after a year [47]. The ‘legacy effect’ was used to explain this observation and may correspond to ‘metabolic memory’ in subjects with T1D [43]. While the exact mechanism of the ‘legacy effect’ is unknown, there is experimental evidence from vascular cells that supports a role for epigenetic changes in the risk of CVD induced by hyperglycaemia [48]. Epigenetic modifications change the expression of genes and pathways associated with endothelial dysfunction (a key step in atherogenesis), and genes involved in metabolic and cardiovascular disease [48]. These epigenetic changes could explain the mechanism by which hyperglycaemia could increase the risk of CVD in subjects with T2D.

## A Balancing Act—Treatment of Type 2 Diabetes and Cardiovascular Disease

The treatment of T2D and CVD may in part reflect the complex relationship between functionally similar pathways that contribute to both diseases but in a mechanistically different way. All new treatments for T2D must prove that they do not increase the risk of CVD outcomes before they can be approved by the Food and Drug Administration [49]. Thiazolidinedione derivatives, used to reduce blood glucose by improving hepatic and peripheral tissue utilisation of glucose, have been associated with adverse cardiovascular outcomes [50] whilst statins, that reduce LDL-C levels, reduce the risk of CVD but increase the risk of T2D [51, 52]. Identifying adverse drug effects before stage 3 clinical trials could reduce the costs of drug development and attrition rates. Genetic studies and MR analyses can be used to investigate the potential outcomes of pharmacological interventions where a suitable genetic instrument is available [53, 54].

A missense variant in *glucagon-like peptide 1 receptor* (*GLP1R*), used to mimic the effects of GLP1R agonists, was associated with reduced risk of CAD, thus providing some evidence that GLP1R agonists are unlikely to be associated with increased cardiovascular risk [53]. The clinical trials for Liraglutide and Semaglutide (two GLP1R agonists) both showed reduced risk of cardiovascular end points in the treatment groups, which support the finding of the MR analysis [53, 55, 56]. Variants in the gene encoding *β-Hydroxy β-methylglutaryl-CoA* (*HMG-CoA*), the target of statins, were associated with reduced cardiovascular risk and with a slight increase in T2D risk [57], which agrees with the trial data on statin use. Proprotein convertase subtilisin/kexin type 9 (PCSK9) inhibitors are a new class of lipid-lowering drugs to reduce the risk of CVD. MR studies of variants in *PCSK9* are associated with reduced risk of CVD but increased risk of T2D, indicating that PCSK9 inhibitors may have similar effects on T2D risk to those seen for statins [54]. The application of MR analyses to drug intervention studies is restricted by how well the genetic instrument mimics the pharmacological intervention and cannot predict off-target drug effects. They can be used with other data sources to accumulate evidence for drug development. There are many omics data sources that are publicly available that can be combined with findings from GWAS, such as eQTL data from GTEx [58] or functional regulatory data from Epigenome RoadMap [59] and a large amount of phenotypic and genetic data available from large population studies, such as the UK Biobank (http://www.ukbiobank.ac.uk/). These data sources can be used to identify features that predict which subjects with T2D are at higher risk of developing cardiovascular complications. There are projects underway using these types of data to improve treatment for cancer: patient data are used to identify digital twins to predict disease progression and identify factors that are important for disease progression [60]. Identification of at-risk individuals can be used to improve clinical trial design but also to enhance treatment regimens, which may open new avenues for treatments.

Although numerous longitudinal studies have highlighted several biomarkers that are likely to be on the causal pathway to T2D and CVD [61], the long pre-clinical phase of both T2D and CVD mean that may not be predictive of disease. In recent years, the number of GWAS of circulating biomarkers has expanded rapidly [62–64]. However, the number of loci typically detected, and the effect sizes on T2D and/or CVD are modest. An MR study of the role of branch chain amino acids (BCAA) in T2D used instruments that explained 5.3–7.5% of the variance in three different BCAA levels [65]. Frequently, findings from these studies have been used to conduct MR studies, although few have been conducted sufficiently stringently as to be robust, and comparisons between studies are hindered by differences in the genetic instruments tested, sample characteristics and power issues. Ongoing GWAS of biomarkers, which include over 500,000 participants, have identified hundreds of new loci for metabolic and anthropometric traits. GRS constructed from larger GWAS will improve the precision of the genetic instruments, based on more accurate estimation of allelic effects. Increased sample size of GWAS studies will also improve power to detect effects of GRS on outcomes even for weaker instruments [66]. In combination, these should improve the robustness of findings from MR studies. Testing causality using these well-powered instruments in large populations with longitudinal data will aid in identifying and ruling out factors that increase the risk of cardiovascular complications.

### Conclusions

There is evidence of shared genetic background underlying T2D and CVD, but it is not reflected in individual locus overlap and is more likely to be due to a handful of shared pathways and risk factors that may have divergent effects on the two diseases. These divergent effects may be reflected in cardiovascular complications because of treatment for hyperglycaemia and increased the T2D risk associated with LDL-C lowering CVD treatments. To advance understanding of the overlap between T2D and CVD, and how shared mechanisms affect each disease, it is essential that causal variation, effector transcripts and effector tissues are identified. Statistical methodology is continuously evolving to maximise the potential of existing genetic datasets, but there is a considerable benefit to understanding disease biology by increasing the sample size of GWAS studies and improving methods for data integration [6•, 67].

## References

[1] Herder C, Karakas M, Koenig W (2011). Biomarkers for the prediction of type 2 diabetes and cardiovascular disease. Clin Pharmacol Ther.

[2] Shore AC, Colhoun HM, Natali A, Palombo C, Ostling G, Aizawa K (2015). Measures of atherosclerotic burden are associated with clinically manifest cardiovascular disease in type 2 diabetes: a European cross-sectional study. J Intern Med.

[3] Thiruvoipati T, Kielhorn CE, Armstrong EJ (2015). Peripheral artery disease in patients with diabetes: epidemiology, mechanisms, and outcomes. World J Diabetes.

[4] Adams HP, Bendixen BH, Kappelle LJ, Biller J, Love BB, Gordon DL (1993). Classification of subtype of acute ischemic stroke. Definitions for use in a multicenter clinical trial. TOAST. Trial of Org 10172 in Acute Stroke Treatment. Stroke.

[5] Daugherty A, Tall AR, Daemen M, Falk E, Fisher EA, Garcia-Cardena G (2017). Recommendation on design, execution, and reporting of animal atherosclerosis studies: a scientific statement from the American Heart Association. ATVB.

[6] • Mahajan A, Taliun D, Thurner M, Robertson NR, Torres JM, Rayner NW, et al. Fine-mapping of an expanded set of type 2 diabetes loci to single-variant resolution using high-density imputation and islet-specific epigenome maps. Nat Genet. 2018 (In press). **This study provides insights into how large GWAS can be used to identify multiple variants associated with T2D and how in combination with other data sources can narrow down the search space for causal variants and transcripts.**

[7] Nikpay M, Goel A, Won HH, Hall LM, Willenborg C, Kanoni S (2015). A comprehensive 1,000 genomes-based genome-wide association meta-analysis of coronary artery disease. Nat Genet.

[8] Scott RA, Lagou V, Welch RP, Wheeler E, Montasser ME, Luan J (2012). Large-scale association analyses identify new loci influencing glycemic traits and provide insight into the underlying biological pathways. Nat Genet.

[9] van Zuydam NR, de Andrade M, Vlachopoulou E, Ahlqvist E, Dahlström E, Salomaa V, et al. A gene-by-environment interaction study of peripheral arterial disease identifies novel loci. Presented at the 66th annual meeting of the American Society of Human Genetics, 18 October 2016, Vancouver. 2016.

[10] Malik R, Traylor M, Pulit SL, Bevan S, Hopewell JC, Holliday EG (2016). Low-frequency and common genetic variation in ischemic stroke: the METASTROKE collaboration. Neurology.

[11] Bulik-Sullivan B, Finucane HK, Anttila V, Gusev A, Day FR, Loh PR (2015). An atlas of genetic correlations across human diseases and traits. Nat Genet.

[12] Zheng J, Erzurumluoglu AM, Elsworth BL, Kemp JP, Howe L, Haycock PC (2017). LD Hub: a centralized database and web interface to perform LD score regression that maximizes the potential of summary level GWAS data for SNP heritability and genetic correlation analysis. Bioinformatics.

[13] Thurner M, van de Bunt M, Torres JM, Mahajan A, Nylander V, Bennett AJ, et al. Integration of human pancreatic islet genomic data refines regulatory mechanisms at Type 2 Diabetes susceptibility loci. eLife 2018;7:e31977. 10.7554/eLife.31977 PMC5828664 29412141

[14] Gaulton KJ, Ferreira T, Lee Y, Raimondo A, Magi R, Reschen ME (2015). Genetic fine mapping and genomic annotation defines causal mechanisms at type 2 diabetes susceptibility loci. Nat Genet.

[15] Scott RA, Scott LJ, Magi R, Marullo L, Gaulton KJ, Kaakinen M (2017). An expanded genome-wide association study of type 2 diabetes in Europeans. Diabetes.

[16] Chan KH, Huang YT, Meng Q, Wu C, Reiner A, Sobel EM (2014). Shared molecular pathways and gene networks for cardiovascular disease and type 2 diabetes mellitus in women across diverse ethnicities. Circ Cardiovasc Genet.

[17] Shu L, Chan KHK, Zhang G, Huan T, Kurt Z, Zhao Y (2017). Shared genetic regulatory networks for cardiovascular disease and type 2 diabetes in multiple populations of diverse ethnicities in the United States. PLoS Genet.

[18] Rivera NV, Carreras-Torres R, Roncarati R, Viviani-Anselmi C, De Micco F, Mezzelani A (2013). Assessment of the 9p21.3 locus in severity of coronary artery disease in the presence and absence of type 2 diabetes. BMC Med Genet.

[19] van Zuydam N, Voight B, Ladenvall C, Strawbridge R, Willems S, Iperen EV (2015). Abstracts of 51st EASD Annual Meeting: a signal near *TMEM170A* is associated with coronary artery disease and SNPs near *IL15RA/IL2RA* and *THY1* may interact with diabetes status to modify the risk of CAD. Diabetologia.

[20] Zhao W, Rasheed A, Tikkanen E, Lee JJ, Butterworth AS, Howson JMM (2017). Identification of new susceptibility loci for type 2 diabetes and shared etiological pathways with coronary heart disease. Nat Genet.

[21] Qi L, Qi Q, Prudente S, Mendonca C, Andreozzi F, di Pietro N (2013). Association between a genetic variant related to glutamic acid metabolism and coronary heart disease in individuals with type 2 diabetes. JAMA.

[22] Wensley F, Gao P, Burgess S, Kaptoge S, Di Angelantonio E (2011). Association between C reactive protein and coronary heart disease: mendelian randomisation analysis based on individual participant data. BMJ.

[23] Davey Smith G, Hemani G (2014). Mendelian randomization: genetic anchors for causal inference in epidemiological studies. Hum Mol Genet.

[24] Ligthart S, de Vries PS, Uitterlinden AG, Hofman A, Franco OH, Group charge Inflammation Working Group (2015). Pleiotropy among common genetic loci identified for cardiometabolic disorders and C-reactive protein. PLoS One.

[25] Jansen H, Loley C, Lieb W, Pencina MJ, Nelson CP, Kathiresan S (2015). Genetic variants primarily associated with type 2 diabetes are related to coronary artery disease risk. Atherosclerosis.

[26] Ross S, Gerstein HC, Eikelboom J, Anand SS, Yusuf S, Pare G (2015). Mendelian randomization analysis supports the causal role of dysglycaemia and diabetes in the risk of coronary artery disease. Eur Heart J.

[27] Larsson SC, Scott RA, Traylor M, Langenberg CC, Hindy G, Melander O (2017). Type 2 diabetes, glucose, insulin, BMI, and ischemic stroke subtypes: Mendelian randomization study. Neurology.

[28] Ahmad OS, Morris JA, Mujammami M, Forgetta V, Leong A, Li R (2015). A Mendelian randomization study of the effect of type-2 diabetes on coronary heart disease. Nat Commun.

[29] Zhu Z, Zheng Z, Zhang F, Wu Y, Trzaskowski M, Maier R (2018). Causal associations between risk factors and common diseases inferred from GWAS summary data. Nat Commun.

[30] Merino J, Leong A, Posner DC, Porneala B, Masana L, Dupuis J (2017). Genetically driven hyperglycemia increases risk of coronary artery disease separately from type 2 diabetes. Diabetes Care.

[31] van Iperen EP, Sivapalaratnam S, Holmes MV, Hovingh GK, Zwinderman AH, Asselbergs FW (2016). Genetic analysis of emerging risk factors in coronary artery disease. Atherosclerosis.

[32] De Silva NM, Freathy RM, Palmer TM, Donnelly LA, Luan J, Gaunt T (2011). Mendelian randomization studies do not support a role for raised circulating triglyceride levels influencing type 2 diabetes, glucose levels, or insulin resistance. Diabetes.

[33] Borges MC, Lawlor DA, de Oliveira C, White J, Horta BL, Barros AJ (2016). Role of adiponectin in coronary heart disease risk: a Mendelian randomization study. Circ Res.

[34] White J, Swerdlow DI, Preiss D, Fairhurst-Hunter Z, Keating BJ, Asselbergs FW (2016). Association of lipid fractions with risks for coronary artery disease and diabetes. JAMA Cardiol.

[35] Beshara A, Cohen E, Goldberg E, Lilos P, Garty M, Krause I (2016). Triglyceride levels and risk of type 2 diabetes mellitus: a longitudinal large study. J Investig Med.

[36] Fall T, Xie W, Poon W, Yaghootkar H, Magi R, Consortium G (2015). Using genetic variants to assess the relationship between circulating lipids and type 2 diabetes. Diabetes.

[37] Voight BF, Peloso GM, Orho-Melander M, Frikke-Schmidt R, Barbalic M, Jensen MK (2012). Plasma HDL cholesterol and risk of myocardial infarction: a Mendelian randomisation study. Lancet.

[38] Haase CL, Tybjærg-Hansen A, Nordestgaard BG, Frikke-Schmidt R (2015). HDL Cholesterol and Risk of type 2 diabetes: a Mendelian randomization study. Diabetes.

[39] Munafò MR, Tilling K, Taylor AE, Evans DM, Davey SG (2018). Collider scope: when selection bias can substantially influence observed associations. Int J Epidemiol.

[40] Paternoster L, Tilling K, Davey Smith G (2017). Genetic epidemiology and Mendelian randomization for informing disease therapeutics: conceptual and methodological challenges. PLoS Genet.

[41] De Rosa S, Arcidiacono B, Chiefari E, Brunetti A, Indolfi C, Foti DP (2018). Type 2 diabetes mellitus and cardiovascular disease: genetic and epigenetic links. Front Endocrinol (Lausanne).

[42] The Diabetes Control and Complications Trial Research Group (1993). The effect of intensive treatment of diabetes on the development and progression of long-term complications in insulin-dependent diabetes mellitus. N Engl J Med.

[43] Kato M, Natarajan R (2014). Diabetic nephropathy—emerging epigenetic mechanisms. Nat Rev Nephrol Nat Rev Nephrol.

[44] Boussageon R, Bejan-Angoulvant T, Saadatian-Elahi M, Lafont S, Bergeonneau C, Kassaï B, et al. Effect of intensive glucose lowering treatment on all cause mortality, cardiovascular death, and microvascular events in type 2 diabetes: meta-analysis of randomised controlled trials. BMJ 2011;343:d4169. 10.1136/bmj.d4169 PMC3144314 21791495

[45] Gerstein HC, Miller ME, Byington RP, Goff DC, Bigger JT (2008). Effects of intensive glucose lowering in type 2 diabetes. N Engl J Med.

[46] The ADVANCE Collaborative Group (2008). Intensive blood glucose control and vascular outcomes in patients with type 2 diabetes. N Engl J Med.

[47] Holman RR, Paul SK, Bethel MA, Matthews DR, Neil HA (2008). 10-year follow-up of intensive glucose control in type 2 diabetes. N Engl J Med.

[48] Pirola L, Balcerczyk A, Tothill RW, Haviv I, Kaspi A, Lunke S (2011). Genome-wide analysis distinguishes hyperglycemia regulated epigenetic signatures of primary vascular cells. Genome Res.

[49] US Food and Drug Administration. Guidance for Industry Diabetes Mellitus—Evaluating Cardiovascular Risk in New Antidiabetic Therapies to Treat Type 2 Diabetes (U.S. FDA, Silver Spring, MD, 2008); http://www.fdagov/downloads/drugs/guidancecomplianceregulatoryinformation/guidances/ucm071627.pdf. 2008.

[50] Kaul S, Bolger AF, Herrington D, Giugliano RP, Eckel RH (2010). Thiazolidinedione drugs and cardiovascular risks: a science advisory from the American Heart Association and American College of Cardiology Foundation. J Am Coll Cardiol..

[51] Aiman U, Najmi A, Khan RA (2014). Statin induced diabetes and its clinical implications. J Pharmacol Pharmacother.

[52] Crandall JP, Mather K, Rajpathak SN, Goldberg RB, Watson K, Foo S, et al. Statin use and risk of developing diabetes: results from the diabetes prevention program. BMJ Open Diabetes Res Care 2017;5(1):e000438. 10.1136/bmjdrc-2017-000438 PMC5652620 29081977

[53] Scott RA, Freitag DF, Li L, Chu AY, Surendran P, Young R (2016). A genomic approach to therapeutic target validation identifies a glucose-lowering *GLP1R* variant protective for coronary heart disease. Sci Transl Med.

[54] Schmidt AF, Swerdlow DI, Holmes MV, Patel RS, Fairhurst-Hunter Z, Lyall DM (2017). PCSK9 genetic variants and risk of type 2 diabetes: a Mendelian randomisation study. Lancet Diabetes Endocrinol.

[55] Marso SP, Bain SC, Consoli A, Eliaschewitz FG, Jódar E, Leiter LA (2016). Semaglutide and cardiovascular outcomes in patients with type 2 diabetes. N Engl J Med.

[56] Marso SP, Daniels GH, Brown-Frandsen K, Kristensen P, Mann JFE, Nauck MA (2016). Liraglutide and cardiovascular outcomes in type 2 diabetes. N Engl J Med.

[57] Swerdlow DI, Preiss D, Kuchenbaecker KB, Holmes MV, Engmann JE, Shah T (2015). HMG-coenzyme A reductase inhibition, type 2 diabetes, and bodyweight: evidence from genetic analysis and randomised trials. Lancet.

[58] GTEx Consortium (2013). The genotype-tissue expression (GTEx) project. Nat Genet.

[59] Chadwick LH (2012). The NIH roadmap epigenomics program data resource. Epigenomics.

[60] Bruynseels K, Santoni de Sio F, van den Hoven J (2018). Digital twins in health care: ethical implications of an emerging engineering paradigm. Front Genet.

[61] Looker HC, Colombo M, Agakov F, Zeller T, Groop L, Thorand B (2015). Protein biomarkers for the prediction of cardiovascular disease in type 2 diabetes. Diabetologia.

[62] Kettunen J, Demirkan A, Wurtz P, Draisma HH, Haller T, Rawal R (2016). Genome-wide study for circulating metabolites identifies 62 loci and reveals novel systemic effects of LPA. Nat Commun.

[63] Dehghan A, Dupuis J, Barbalic M, Bis JC, Eiriksdottir G, Lu C (2011). Meta-analysis of genome-wide association studies in > 80 000 subjects identifies multiple loci for C-reactive protein levels. Circulation.

[64] Ahola-Olli AV, Wurtz P, Havulinna AS, Aalto K, Pitkanen N, Lehtimaki T (2017). Genome-wide association study identifies 27 loci influencing concentrations of circulating cytokines and growth factors. Am J Hum Genet.

[66] Burgess S (2014). Sample size and power calculations in Mendelian randomization with a single instrumental variable and a binary outcome. Int J Epidemiol.

[67] Horikoshi M, Beaumont RN, Day FR, Warrington NM, Kooijman MN, Fernandez-Tajes J (2016). Genome-wide associations for birth weight and correlations with adult disease. Nature.

[65] Lotta LA, Scott RA, Sharp SJ, Burgess S, Luan J, Tillin T (2016). Genetic predisposition to an impaired metabolism of the branched-chain amino acids and risk of type 2 diabetes: a Mendelian randomisation analysis. PLoS Med.

